# Case report and literature review on a large MBC with ulceration

**DOI:** 10.1097/MD.0000000000033131

**Published:** 2023-03-03

**Authors:** Qin-Qin Luo, Na-Na Luo

**Affiliations:** a Traditional Chinese Medicine Department of Wuhan No.1 Hospital, China; b Nuclear Medicine Department of Hubei Cancer Hospital, China.

**Keywords:** case report, MBC, skin ulceration, traditional Chinese medicine

## Abstract

Metaplastic breast cancer (MBC) is a rare tumor of the breast, and skin ulceration of breast tumors is a difficult clinical problem that reduces the patient’s quality of life.

**Patient concerns:**

There are currently no Standard Treatment Guidelines for MBC at present, and the treatment for the skin ulceration of breast tumors is limited in clinics.

**Diagnosis:**

Here, we report the case of a patient with a large MBC and skin ulceration, accompanied by exudation and odor.

**Intervention:**

The combined treatment of albumin paclitaxel and carrelizumab (anti-PD-1 immunotherapy) was effective in reducing the tumor, but it increased the severity of the skin ulceration. After taking traditional Chinese medicine, the skin ulceration healed completely. Then the patient underwent a mastectomy and radiotherapy.

**Outcomes:**

The patient has a high quality of life and was in good condition after the comprehensive treatment.

**Lessons:**

This suggests that traditional Chinese medicine may have a good auxiliary therapeutic effect on the skin ulceration of MBC.

## 1. Introduction

At present, breast cancer is a malignant tumor with the highest incidence in the world, and is also the main cause of cancer-related death in women.^[1]^ Metaplastic breast cancer (MBC) is a rare pathological type of breast cancer with obvious heterogeneity, and its morbidity is <1% of all breast cancers.^[2,3]^ The most common clinical symptom of MBC is a painless large breast lump. When breast tumor cells proliferate and invade the skin, skin ulceration can occur, which is often accompanied by bleeding, pain, exudation, and odor. For serious skin ulcerations, skin grafting is necessary. Skin ulceration is a difficult clinical problem, which not only reduces patients’ quality of life (QoL) but also creates barriers to radical mastectomy.

In this study, we observed the perfect healing of skin ulceration in a patient with a large MBC following the adjuvant therapy of traditional Chinese medicine (TCM).

## 2. Case report

**Figure 1.**
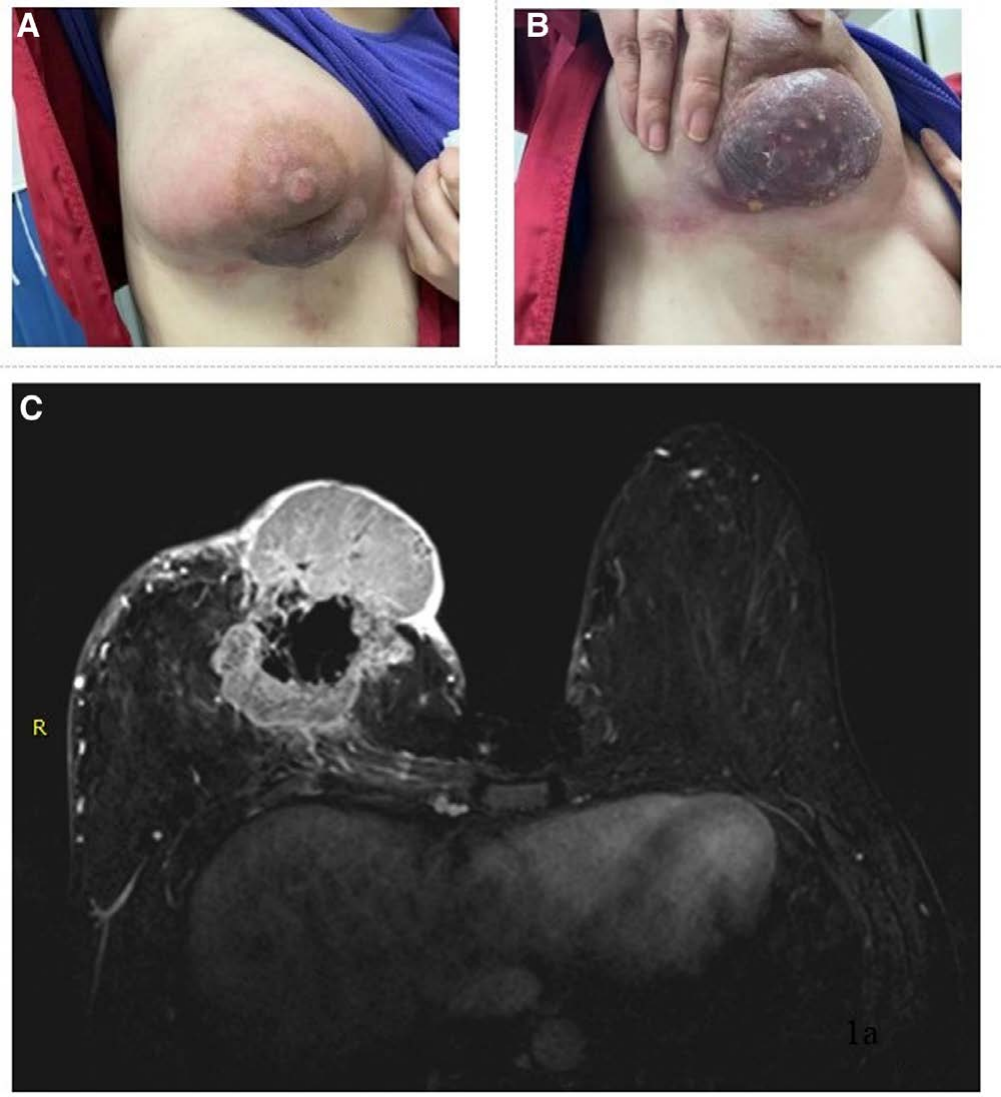
(A and B) Appearance of the breast mass before the treatment. (C) Enhanced MRI of the breast before the treatment. MRI = magnetic resonance imaging.

A young woman in her 30s visited a previous institute in April 2022 because she had noticed a right breast lump growing rapidly within a few months (Fig. 1A and B). Breast biopsy revealed a mixed tissue of invasive glandular carcinoma and multifocally distributed metaplastic carcinoma. Immunohistochemistry of the tumor confirmed the following: Glandular carcinoma: HER2 1+, estrogen receptor-negative, progesterone receptor (+,2%), and Ki-67 = 60%; Metaplastic carcinoma: HER2-negative, estrogen receptor-negative, progesterone receptor-negative, and Ki-67 = 40%. Enhanced magnetic resonance imaging of the breast revealed a right breast tumor (87 × 66 × 63 mm) (Fig. 1C). Computed tomography of the head, chest, and abdomen, and whole-body bone scan indicated no distant metastasis. The results of the blood tests were normal, and the levels of carcinoembryonic antigen reached 46.0 ng/mL. The large breast tumor was painless and the small skin ulceration was accompanied by exudation and odor, which decreased her QoL. And her Eastern Cooperative Oncology Group performance score was 0.

**Figure 2.**
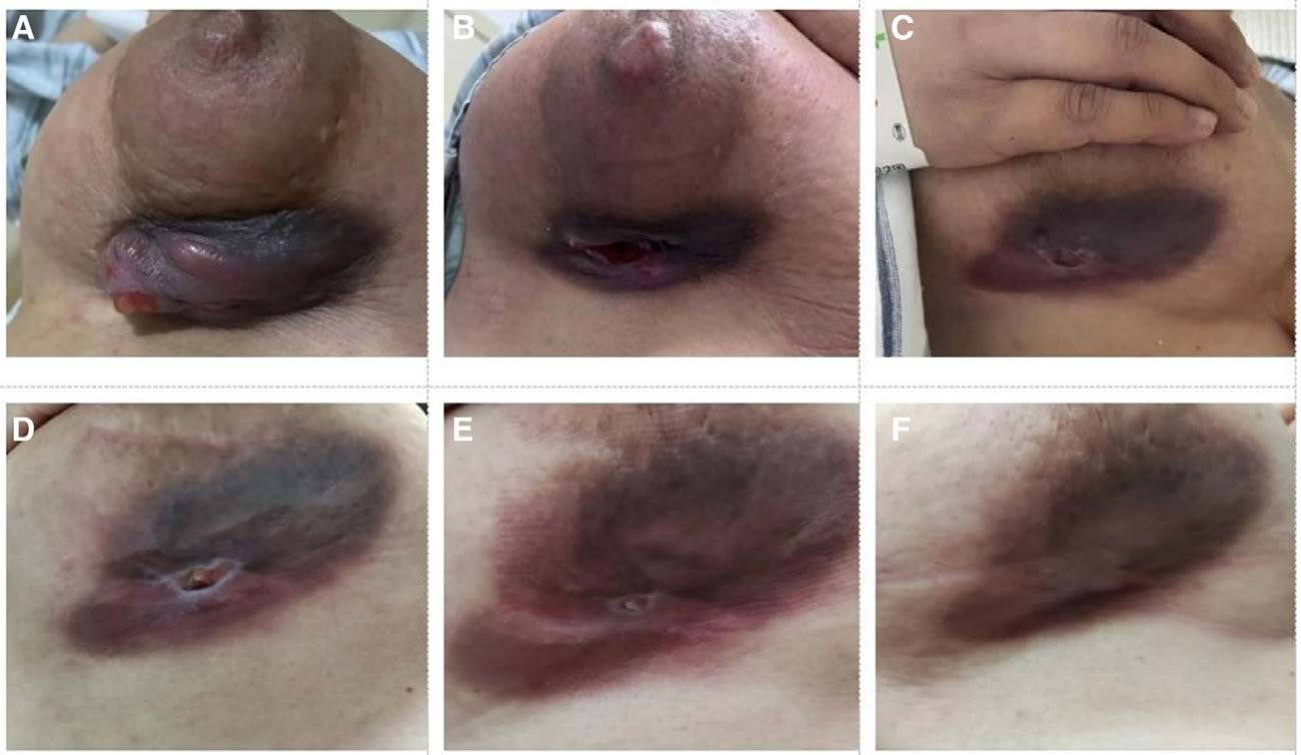
The change of the patient’s breast skin ulcer wound during the comprehensive treatment. (A) After the first cycle of albumin paclitaxel and Carrelizumab(27/06/2022). (B) After the second cycle of albumin paclitaxel and Carrelizumab(11/07/2022). (C) After taking TCM(25/07/2022). (D) After taking TCM(29/07/2022). (E) After taking TCM(08/08/2022). (F) After taking TCM(26/08/2022). TCM = traditional Chinese medicine.

**Figure 3.**
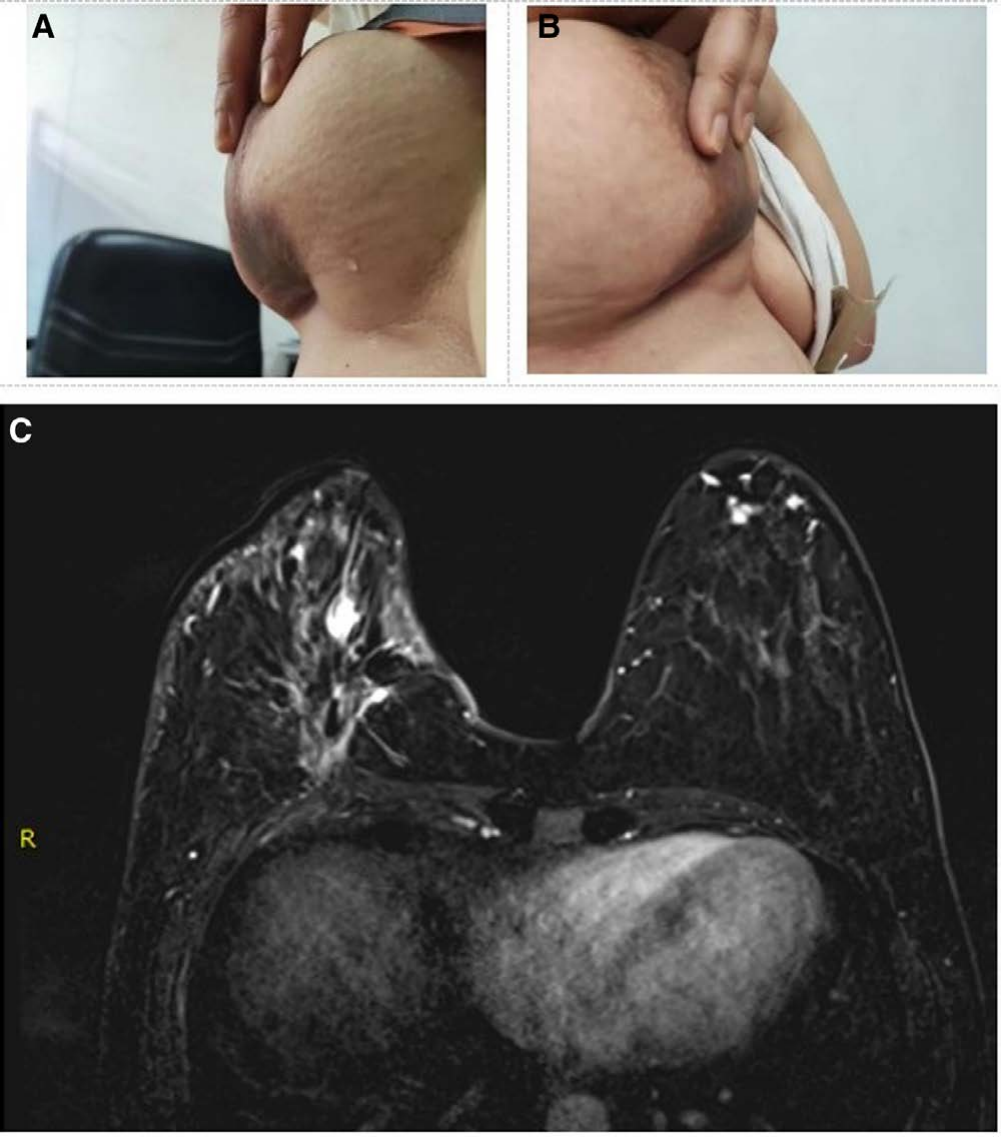
(A and B) Appearance of the breast after the comprehensive treatment. (C) Enhanced MRI of the breast after the comprehensive treatment. MRI = magnetic resonance imaging.

Initially, she received 3 cycles of epirubicin and cyclophosphamide chemotherapy, but the tumor did not reduce. Albumin paclitaxel and carrelizumab were administered on June 13, 2022. The tumor size was remarkablely reduced after the first cycle of chemotherapy (Fig. 2A). Therefore, the patient completed the other 5 cycles, and the exact times of the 6 cycles were (June 13, 2022, June 28, 2022, July 7, 2022, July 27, 2022, August 10, 2022, August 23, 2022). After the second cycle, the large neoplasm shrank remarkably, while the skin ulcers fused and became more serious, which was bleeding and odoriferous (Fig. 2B). She visited our institute and sought help from the TCM (July 15, 2022). According to her signs and symptoms, we provided a Chinese herbal medicine decoction (200 ml, twice a day), which had the effect of reinforcing qi and nourishing blood, the main Chinese herbs are described in Table 1. The skin ulceration healed gradually after the patient took the decoction (Fig. 2C). During the next 3 cycles of chemotherapy, she insisted on taking a Chinese herbal medicine decoction, which was adjusted slightly according to her symptoms, and the skin ulceration was gradually healed (Fig. 2D and E). After the last cycle of chemotherapy, the skin ulceration healed completely without any scarring (Fig. 2F). The primary lump disappeared and skin retraction was observed (Fig. 3A and B). Enhanced magnetic resonance imaging of the breast revealed that the breast lump had completely faded away (Fig. 3C). The patient’s QoL was improved and she was confident in accepting the following treatment since her skin ulceration began to heal. After the chemotherapy, the patient underwent mastectomy on September 12, 2022. Pathological examination revealed axillary sentinel lymph nodes metastasis (2/4) (ypT0N1M0). The surgical wound healed well and radiotherapy was completed. The patient was in good condition and was receiving treatment with carrelizumab (once every 3 weeks).

**Table 1:** The prescription of Chinese herb.

Effect	Chinese herbs
Invigorating spleen and replenishing energy	(Astragalus) 40 g, (Radix codonopsis)15 g, (Atractylodes macrocephala)10 g, (Ganoderma lucidum)15 g
Nourishing yin and supplementing blood	(Angelicae) 6 g, (Ligustrum lucidum Ait) 15 g, (Polygonatum Kingianum)15 g, (Semen Ziziphi Spinosae) 15 g
Regulating qi and removing moisture	(Pinellia ternate) 6 g, (Citrus) 6 g, (Poria cocos)15 g, (Semen coicis) 30 g

## 3. Discussion and review

MBC is a rare and unique type of breast cancer. According to the latest World Health Organization classification criteria for breast and female reproductive system tumors of World Health Organization, MBC can be divided into 6 subtypes: squamous cell carcinoma (SCC), spindle cell carcinoma, low-grade SCC, mesenchymal differentiated metaplasia carcinoma, fibromatosis metaplasia carcinoma, and mixed metaplasia carcinoma. Currently, the Vogts and Norris classification methods are widely used in the classification of MBC, which can be divided into 5 subtypes: spindle cell carcinoma, SCC, carcinosarcoma, stromal carcinoma, and osteoclast giant cell carcinoma.^[3]^ The most common subtypes in the western world are spindle cell carcinoma and SCC in the eastern world. The pathology of this patient’s tumor was a heterogeneous tumor mixed with metaplastic carcinoma and adenocarcinoma, which is extremely rare in the clinic. The pathology of metaplastic cancer is SCC. The most common sign of MBC is painless breast lumps, but the incidence rate of lymph node metastasis is relatively low.^[4–6]^ The pathological features of MBC are usually triple negative.^[7–9]^ In this case, the breast tumor was large but painless and the pathological features were consistent with those reported in the literature.

Regarding the prognosis of metaplasia of breast cancer, it is generally believed that compared with triple-negative breast cancer, it has a worse prognosis because it is more invasive and more likely to have recurrence and distant metastasis. As most patients with MBC are triple negative, they cannot benefit from endocrine therapy or postoperative targeted therapy. Moreover, MBC has low sensitivity to chemotherapy. Although anthracyclines have been recommended to treat this special cancer in a previous study,^[9]^ there is still no standard systematic treatment for MBC. In this case, there was no change in the breast lump after 3 cycles of epirubicin and cyclophosphamide chemotherapy (epirubicin + cyclophosphamide), which may be partly due to the heterogeneity of the patient’s tumor. The combination of albumin paclitaxel and immunotherapy had performed a positive effect, suggesting that this treatment may be an effective method for heterogeneous breast cancer with metastatic carcinoma.

Skin ulceration is a difficult clinical problem because of the continuous enlargement of breast cancer. Cancerous skin ulceration is often accompanied by bleeding, exudation, pain, and odor, which have a negative effect on the patients’ mental health and QoL. In severe cases, the skin ulceration cannot heal for a long time. Eventually, the necrotic tissue easily leads to sepsis due to bacteria or other infections, as well as death due to the rapid growth of tumors. Skin ulceration of breast cancer not only seriously affects patients’ mental health and QoL seriously, but also makes the treatment difficult. At present, anti-tumor therapies, such as chemotherapy, endocrine therapy, immunotherapy, targeted therapy, and surgery are the main treatment for skin ulceration. However, some patients with large tumors and severe ulcerations must undergo autologous skin grafting after surgical resection.^[10]^ In addition to tumor cell proliferation, long-term chemotherapy, radiation therapy, laser therapy, and other treatments can also lead to or aggravate skin ulceration in breast tumors. For example, paclitaxel has been reported to cause skin ulceration.^[11]^ Moreover, experimental studies have shown that paclitaxel can lead to skin ulcers in mice, and the severity of skin ulcers is positively correlated with the dose of paclitaxel.^[12]^

At present, there are a few clinical studies on breast cancer with skin ulceration, and some case reports have been published. For example, a 31-year-old patient, who was diagnosed with advanced breast cancer with a skin ulcer (T4bN3bM0), received 4 cycles of cyclophosphamide, epirubicin and 5-FU chemotherapy and 4 cycles of paclitaxel combined with Target of Rapamycin. The patient achieved partial remission, and her QoL significantly improved. There was no recurrence 1 year after radical mastectomy.^[13]^ A 68-year-old patient with advanced breast cancer and a huge skin ulcer received long-term combined treatment of trastuzumab (>400 times), aromatase inhibitors, and anti-cancer drugs. She achieved a progression-free survival of >9 years.^[14]^ The QoL of an elderly patient with a huge breast tumor is seriously affected by a severe skin ulcer. After comprehensive treatment including chemotherapy, radiotherapy, surgery, and skin grafting, the breast tumor was removed and the serious skin ulcer was repaired. Although the tumor recurred 1 year later, the patient’s QoL was significantly improved.^[15]^ Some scholars have used paclitaxel combined with bevacizumab as neoadjuvant chemotherapy to treat 2 cases of large breast tumors with skin ulceration. The skin ulcer was reduced and their QoL was improved after prolonged chemotherapy. They have successfully performed radical surgery at last.^[16]^ In foreign countries, systemic anti-cancer therapy (such as chemotherapy) is the main treatment for breast cancer with skin ulceration, whereas, there are few local treatments for skin ulceration.

In China, systemic anti-cancer therapy remains the main treatment for breast cancer patients with skin ulceration, and local treatment for skin ulceration has also been explored. For example, a scholar applied a 25% 5-FU (750 mg/day) solution wet dressing to treat a patient with a skin ulcer in a huge breast tumor, accompanied by systemic chemotherapy treatment. The skin ulcer was significantly improved after 2 cycles. The patient underwent radical surgery, and there was no recurrence or metastasis during the follow-up period of >2 years.^[17]^ Han Yu^[18]^ used local tissue implant radiotherapy to treat a huge skin ulcer in a breast cancer patient who had not received a therapeutic effect from the comprehensive treatment. Local radiotherapy reduced the tumor load in a relatively short time and improved the patient’s QoL. Wang Haimei^[19]^ treated 30 breast cancer patients with skin ulceration using exterior coating nano-realgar powder, which was made from a Chinese herbal medicine realgar. Compared with the control group (5% cyclophosphamide solution wet dressing), the treatment group (nano-realgar exterior coating) promoted the healing of skin ulcers more effectively after 3 months of treatment, suggesting that the local application of new type of Chinese medicine may be an efficient method for the treatment of breast cancer with skin ulceration.

In this case, the tumor was significantly reduced after the treatment with albumin paclitaxel and carilizumab. However, the skin ulcers gradually fused into a larger wound with bleeding and odor, which reduced the patient’s QoL. Almost all the doctors told the patient that the skin ulcer wound not only could not heal but would become more serious during chemotherapy. The skin ulcer wound gradually healed after taking Chinese medicine, which greatly encouraged the patient. She told us that TCM was wonderful and that the magical efficacy increased her confidence in receiving the following treatment.

In this case, the prescription is based on the Danggui Buxue Decoction and Sijunzi Decoction. Danggui Buxue Decoction is composed of Astragalus and Angelicae, which is a classic formula for nourishing qi and blood. It has been confirmed that the main active ingredients of Astragalus and Angelicae can repair the oxidative damage of vascular endothelial cells and play a role in inflammation.^[20,21]^ It has been found that Astragalus and Angelicae are the 2 most frequently used herbs in the treatment of ecthyma and diabetic foot ulcers by the method of data mining.^[22,23]^ In the diabetic foot ulcer wound healing rat model, Danggui Buxue Decoction may promote vascular regulatory molecules expression of NO by increasing inducible NOS expression, which may promote vascular endothelial growth factor (VEGF) expression and accelerate wound healing simultaneously.^[24]^ Danggui Buxue Decoction decoction can reduce the inflammatory response in diabetic foot ulcer rat models and promote neovascularization to accelerate wound healing.^[25]^ Sijunzi Decoction is a classic prescription for replenishing qi in clinics and is composed of Radix Codonopsis, Atractylodes macrocephala, Poria cocos, and Licorice. Studies have shown that Sijunzi Decoction can promote the expression of the Klotho gene to inhibit the atrophy of the skin and subcutaneous adipose tissue, and improve the free radical metabolism by increasing the activity of superoxide dismutase, thereby protecting the skin.^[26,27]^ In vivo, Sijunzi Decoction can accelerate angiogenesis and promote the growth of granulation tissue and wound Eepithelialization, thereby promoting wound healing by accelerating the proliferation of human fibroblasts and the expression of EGF, TGF-β, and VEGF.^[28]^ In a mouse model of refractory ulcers, the Sijunzi Decoction may promote the growth of granulation tissue, and increase the expression of VEGF to accelerate wound angiogenesis, thus promoting wound healing.^[29,30]^

Because it is rare and highly invasive, prospective research on MBC is rare. Most of the published data are derived from retrospective studies, and there are currently no Standard Treatment Guidelines for MBC at present. Therefore, it is still necessary to formulate the most appropriate treatment plan according to patients’ own conditions in clinical practice. In this case, the patient’s breast tumor was significantly reduced after the combination of albumin paclitaxel and immunotherapy, and the skin ulcer healed well after the adjuvant administration of TCM, suggesting that TCM may have a good adjuvant effect on patients with breast cancer accompanied by skin ulceration.

## Acknowledgments

We would like to thank all the staff and nurses for their kind cooperation. We would also like to thank the patient.

## Author contributions

**Resources:** Qin Qin Luo, Na Na Luo.

**Supervision:** Qin Qin Luo, Na Na Luo.
